# Directed Differentiation of Human Pluripotent Stem Cells toward Skeletal Myogenic Progenitors and Their Purification Using Surface Markers

**DOI:** 10.3390/cells10102746

**Published:** 2021-10-14

**Authors:** Nasa Xu, Jianbo Wu, Jose L. Ortiz-Vitali, Yong Li, Radbod Darabi

**Affiliations:** 1 Center for Stem Cell and Regenerative Medicine (CSCRM), The Brown Foundation Institute of Molecular Medicine for the Prevention of Human Diseases (IMM), University of Texas Health Science Center at Houston, Houston, TX 77030, USA; nasa.y.xu2@gmail.com (N.X.); Jianbo.Wu@uth.tmc.edu (J.W.); jose.ortizvitali@gmail.com (J.L.O.-V.); 2 Department of Orthopaedic Surgery, BioMedical Engineering, Western Michigan University Homer Stryker M. D. School of Medicine (WMed), Kalamazoo, Michigan, MI 49008, USA; Yong.Li@med.wmich.edu

**Keywords:** human iPSCs, skeletal muscle differentiation, muscle progenitors, stem cells, differentiation method

## Abstract

Advancements in reprogramming somatic cells into induced pluripotent stem cells (iPSCs) have provided a strong framework for in vitro disease modeling, gene correction and stem cell-based regenerative medicine. In cases of skeletal muscle disorders, iPSCs can be used for the generation of skeletal muscle progenitors to study disease mechanisms, or implementation for the treatment of muscle disorders. We have recently developed an improved directed differentiation method for the derivation of skeletal myogenic progenitors from hiPSCs. This method allows for a short-term (2 weeks) and efficient skeletal myogenic induction (45–65% of the cells) in human pluripotent stem cells (ESCs/iPSCs) using small molecules to induce mesoderm and subsequently myotomal progenitors, without the need for any gene integration or modification. After initial differentiation, skeletal myogenic progenitors can be purified from unwanted cells using surface markers (CD10^+^CD24^−^). These myogenic progenitors have been extensively characterized using in vitro gene expression/differentiation profiling as well as in vivo engraftment studies in dystrophic (mdx) and muscle injury (VML) rodent models and have been proven to be able to engraft and form mature myofibers as well as seeding muscle stem cells. The current protocol describes a detailed, step-by-step guide for this method and outlines important experimental details and troubleshooting points for its application in any human pluripotent stem cells.

## 1. Introduction

Human induced pluripotent stem cells (hiPSCs) serve as an excellent model system to study disease phenotype and mechanism, identify therapeutic targets and develop stem cell-based therapies for many degenerative disorders [1,2]. Due to their ease of derivation and reprogramming from somatic cells, pluripotency and differentiation potential as well as autologous source, iPSCs are an excellent candidate for regenerative medicine [3,4]. Therefore, many efforts have been made to improve iPSC reprogramming and lineage-specific differentiation methods [5,6,7,8,9,10,11,12,13]. The main purpose of these efforts is to provide safe cell prep techniques useful for translational medicine, such as integration-free methods for iPSC reprogramming to eliminate the risk of mutagenesis or the production of various tissue progenitors by mirroring natural developmental signaling through directed differentiation methods [4,6].

In this regard, our group has a long-term interest in generation of safe and scalable skeletal muscle progenitors from iPSCs useful for disease modeling or regenerative applications [14,15,16]. Recently, by developing a knock-in double reporter human ESC cell line for PAX7 and MYF5, we were able to screen important governing pathways to develop a directed differentiation method for derivation of skeletal myogenic progenitors from human pluripotent stem cells (hPSCs) [17,18,19]. These efforts led to the development of an efficient and short-term differentiation and purification strategy for the isolation of skeletal myogenic progenitors. This method can be applied to any human ESC or iPSC without the need for any gene modification or overexpression but rather through only providing natural developmental cues during early phases of embryogenesis [19]. In this protocol, by the initial induction of presomitic mesoderm using WNT activation, followed by somite/myotome induction through TGF-β and BMP inhibition, skeletal myogenic progenitors can be isolated using surface markers (CD10^+^CD24^−^). This method allows for a short-term (2 weeks) and efficient induction of skeletal myogenic program (45–65% of the cells) in hiPSCs [19]. In our recent reports, these cells have been fully characterized for myogenic proliferation and differentiation potential using in vitro transcriptomic profiling and terminal differentiation assays [19]. In addition, our in vivo studies have demonstrated regeneration potential of hiPSC-derived myogenic progenitors in dystrophic (mdx) and volumetric muscle loss (VML) mouse models [19,20].

In order to facilitate the application of this method, here we describe a step-by-step differentiation protocol to derive skeletal myogenic progenitors from any hPSC line (ESCs/iPSCs) using the abovementioned method. Important practical and troubleshooting points have also been discussed to provide a detailed guideline for this differentiation method. Successful implementation of this protocol facilitates the application of hiPSCs for disease phenotyping, drug screening and the development of therapeutic strategies for skeletal muscle disorders and dystrophies by other investigators.

## 2. Materials

Appropriate personal protective equipment (PPE) should always be used when working with cells. All necessary media and solutions should also be prepared with aseptic techniques under a class II type A2 biosafety cabinet. We recommend wiping gloves and any reagent containers with 70% ethanol solution before transferring into the biosafety cabinet. All reagents used in this protocol, including the chemicals and solvents such as water and PBS, should be free from pathogens, cell culture grade and sterile. Additionally, we recommend supplementing all cell growth media with penicillin-streptomycin (Pen/Strep) to avoid bacterial contamination. Throughout expansion and differentiation, all cell lines need to be grown in 5% CO_2_ at 37 °C. Biohazard waste materials used in this protocol must be properly disposed of in accordance with your institution’s guidelines.

### 2.1. Reagents, Medium and Supplies Needed for Expansion of hPSCs

1. Corning^®^ Matrigel^®^ hESC-qualified matrix, LDEV-free (Thermo Fisher Scientific, Waltham, Massachusetts, MA, USA, 354277) for coating of plates or flasks before seeding of hESCs or hiPSCs.
2. mTeSR™1 cGMP, Feeder-Free Maintenance Medium for human ES and iPS Cells (Stem Cell Technologies, Vancouver, BC, V6A 1B6, Canada, 85850).
3. Accutase™ Cell Detachment Solution (Stem Cell Technologies, 07922).
4. 10 mM ROCK Inhibitor (Y-27632) (Selleck Chemicals, Houston, TX, USA, S1049).
5. PBS, HyClone™ Dulbecco’s Phosphate-Buffered Saline Solution without calcium, magnesium (GE Healthcare Life Sciences, Chicago, IL, USA, SH30028.02).
6. Corning™ Costar™ Flat Bottom Cell Culture Plates (Thermo Fisher Scientific).
7. Nunc T25 and T75 cell culture-treated polystyrene flasks with filter caps (Thermo Fisher Scientific).

### 2.2. Reagents, Medium and Supplies Needed for Skeletal Myogenic Differentiation of hPSCs (Stage I and Stage II)

1. IMDM (1× Iscove’s Modified Dulbecco’s Medium with L-Glutamine and 25 mM HEPES (Thermo Fisher Scientific, 12440053)
2. Horse Serum, heat inactivated (Thermo Fisher Scientific, 26050088)
3. CHIR99021 (R&D Systems, Minneapolis, MN, USA, 4423/10)
4. SB431542 (Selleck Chemical LLC, Houston, TX, USA, S1067)
5. Recombinant Human EGF (PeproTech Inc., East Windsor, NJ, USA, AF-100-15)
6. Insulin solution human (Sigma-Aldrich, Saint louis, MO, USA, I9278)
7. Dexamethasone (Stem Cell Technologies, 72092)
8. L-Ascorbic acid, suitable for cell culture (Sigma-Aldrich, A4544)
9. Gibco^®^ GlutaMAX™ Supplement (Thermo Fisher Scientific, 35050079)
10. Penicillin-Streptomycin, 5000 U/mL (Thermo Fisher Scientific, 15070063)
11. LDN193189 (Stemgent, Cambridge, MA, USA, 04-0074)
12. Recombinant Human HGF (PeproTech Inc., USA, 100-39H)
13. Recombinant Human FGF (PeproTech Inc., AF-100-18B)
14. Recombinant Human IGF-1 (PeproTech Inc., 100-11)
15. Gibco™ KnockOut™ Serum Replacement (Thermo Fisher Scientific, 10828010)
16. Accutase™ Cell Detachment Solution (Stem Cell Technologies, 07922)
17. 10 mM ROCK Inhibitor (Y-27632) (Selleck Chemicals, S1049)

### 2.3. Reagents, Buffers and Supplies Needed for Cell Sorting Using Fluorescence-Activated Cell Sorting (FACS)

1. FACS staining buffer: PBS buffer, HyClone™ Dulbecco’s Phosphate-Buffered Saline Solution without calcium, magnesium (GE Healthcare Life Sciences, SH30028.02) supplemented with 2% Gibco™ Fetal Bovine Serum (Thermo Fisher Scientific, A4766801)
2. FACS sorting buffer with DAPI: PBS, HyClone™ Dulbecco’s Phosphate-Buffered Saline Solution without calcium, magnesium (GE Healthcare Life Sciences, SH30028.02) supplemented with 2% Gibco™ Fetal Bovine Serum (Thermo Fisher Scientific, A4766801) and 0.1 μg/mL DAPI Invitrogen™ (Thermo Fisher Scientific, D1306)
3. Primary blocking antibody: Invitrogen FC Receptor Binding Inhibitor Polyclonal Antibody, eBioscience™ (Thermo Fisher Scientific, 14-9161-73)
4. CD10 antibody: APC Mouse antihuman CD10 antibody (BioLegend, San Diego, CA, USA, 312210)
5. CD24 antibody: BV421 Mouse antihuman CD24 antibody (BD Biosciences, Hackensack, NJ, USA, 562789)
6. Falcon^®^ 5 mL Round Bottom Polystyrene Test Tube, with Cell Strainer Snap Cap (Corning Inc., Corning, NY, USA, 352235)
7. Accutase™ Cell Detachment Solution (Stem Cell Technologies, 07922)
8. 10 mM ROCK Inhibitor (Y-27632) (Selleck Chemicals, S1049)
9. PBS, HyClone™ Dulbecco’s Phosphate Buffered Saline Solution without calcium, magnesium (GE Healthcare Life Sciences, SH30028.02)

### 2.4. Reagents, Medium and Supplies Needed for Terminal Differentiation of hPSCs into Myotubes (Stage III)

1. IMDM (1× Iscove’s Modified Dulbecco’s Medium with L-Glutamine and 25 mM HEPES (Thermo Fisher Scientific, 12440053)
2. Gibco™ KnockOut™ Serum Replacement (Thermo Fisher Scientific, 10828010)
3. Recombinant Human IGF-1 (PeproTech Inc., 100-11)
4. Penicillin-Streptomycin, 5000 U/mL (Thermo Fisher Scientific, 15070063)

### 2.5. Reagents and Supplies Needed for Immunostaining of Myotubes

1. PBS, HyClone™ Dulbecco’s Phosphate-Buffered Saline Solution without calcium, magnesium (GE Healthcare Life Sciences, SH30028.02)
2. 4% Paraformaldehyde (PFA) Solution: 4% PFA in PBS (ACROS Organics, USA, 41678-5000)
3. Purified Mouse Anti-Myogenin antibody (BD Biosciences, USA, 556358)
4. Mouse Anti-Myosin heavy chain antibody (Developmental Studies Hybridoma Bank, Iowa City, IA, USA, MF 20)

## 3. Methods

For maintenance and culturing of hPSCs, all culture plates and flasks should be coated with a Matrigel solution to promote cell attachment and differentiation of the cells.

### 3.1. Matrigel Coating of Plates and Flasks

1. Thaw the Matrigel vial by submerging it in ice in a 4 °C refrigerator overnight. Next, swirl the vial and divide into aliquots of 135–175 μL according to the dilution factor provided on the certificate of analysis for each lot. Store aliquots at −20 °C. If a different hESC-qualified Matrigel is used, refer to the respective certificate of analysis for storage and aliquot information.
2. For coating plates and flasks, thaw one Matrigel aliquot on ice. Each vial is enough for making 12.5 mL of the coating solution. It is important to thaw on ice because Matrigel begins to form a gel at about 10 °C. Once thawed, thoroughly mix Matrigel with 12.5 mL of cold DMEM/F12 medium. If more Matrigel coating is needed, thaw accordingly. Use the following amount of coating medium for each type of plate or flask (Table 1).
3. Quickly transfer the appropriate volume of cold Matrigel mixture into a plate or flask and ensure the coating solution has covered the entire surface of the plate or flask by gentle tilting it.
4. Incubate the plate or flask under the cell culture hood for 1 h at room temperature. If the plate or flask is not used immediately, it can be sealed with Parafilm® and stored at 4 °C for up to 1 week.

### 3.2. Thawing, Expansion and Passaging of hPSCs (ESCs/iPSCs)

#### 3.2.1. Thawing and Expansion of hPSCs

We generally freeze and thaw hPSCs in 6-well plate format. This means freezing the cells from 1 well in 4–6 cryovials and thawing each back into 1 well. This ratio might vary depending of each PSC line. Therefore, each investigator has to determine the optimal freeze–thaw ratio for each PSC line. Additionally, ROCK inhibitor (see Section 2.1) is used in a 1:1000 ratio to mTeSR medium (1 μL ROCK inhibitor/mL of medium) throughout this protocol to increase cloning efficiency and cell survival, which is especially important when thawing and passaging hPSCs.

1. Prior to thawing hPSCs, coat desired plates or flasks with Matrigel, as detailed above (see Section 3.1). As mentioned above, we generally use a 6-well plate for initial thawing of hPSCs. However, it can be scaled up to different plates or flasks.
2. Warm the complete mTeSR medium (see Section 2.1) in room temperature for 30 min to 1 h.
3. Partially thaw one vial of hPSCs from −80 °C by placing it into a 37 °C water bath with gentle shaking. When only a small piece of ice (about 5 mm in diameter) remains, remove the vial from the water bath and mix gently with 5 mL of complete mTeSR medium by pipetting up and down about 4–6 times in a 15 mL conical tube. Be gentle to not break the cell aggregates too much.
4. Centrifuge the cell suspension at 200–300× *g* at room temperature for 5 min. During this time, prepare the resuspension medium by thoroughly mixing 2 mL of the complete mTeSR medium with 2 μL of ROCK inhibitor.
5. Aspirate the supernatant with a sterile Pasteur pipette. This should be performed carefully to avoid removing the remaining cell pellet.
6. Gently break apart the cell pellet with the 2 mL resuspension medium by pipetting up and down about 4–6 times to separate cell clumps. Make sure the pellet is not broken down into single cells as this reduces cell survival and plating efficiency.
7. Aspirate the excess Matrigel coating solution from the well using a sterile glass pasture pipette. Add the cells in resuspension medium by pipetting against the side wall of the plate to avoid scratching off the coating.
8. Transfer the cells into an incubator. Ensure the cells have been evenly distributed across the plate by moving it in a straight side-to-side and up-and-down motion when placing them into the incubator.
9. Allow the cells to attach to the plate or flask by incubating it in 37 °C at 5% CO_2_ overnight. (see Note 1)
10. The next day (Day 1), check the cells using an inverted light microscope at 4× magnification. After one day of culture, hPSCs should be attached and forming small colonies. If the initial plating density was low or in case of low survival, colonies may develop later.
11. Remove unattached/dead cells by changing the medium. (see Notes 2 and 3) The cell culture medium (complete mTeSR medium without ROCK inhibitor) should be replaced every day. To change the medium, gently shake the plate side to side to float dead cells and aspirate using a sterile glass pasture pipette. Next, gently add fresh warmed medium to the side wall of the plate or flask to prevent cell detachment.

#### 3.2.2. Passaging of hPSCs

Depending on the seeding density, iPSC cultures are usually passaged in about 5–7 days. This generally happens when the colonies are large and beginning to merge. We recommend expansion and passaging of the freshly thawed hPSCs at least once before starting the myogenic differentiation protocol to have the cells in their optimal growth rate.

1. Prior to passaging hPSCs, ensure that the required plates or flasks for subculture are coated with Matrigel, as detailed above (see Section 3.1).
2. Warm the complete mTeSR medium and Accutase at room temperature for 30 min to 1 h.
3. Aspirate the used medium from the plate or flask and add PBS (same volume of used medium) to wash out any leftover medium or dead cells.
4. Aspirate the PBS wash and add enough Accutase to cover the surface of the plate or flask. Tilt the plate or flask to ensure complete coverage. We recommend the following volumes of Accutase for each type of plate or flask (Table 2):
5. Incubate the Accutase and cells in a 37 °C incubator for 3 min. After incubation, tap the plate or flask to make sure the cell clumps have detached from the surface. (see Note 4)
6. After confirming cell detachment, add complete mTeSR medium to the well or plate to stop the enzymatic reaction. The volume of complete mTeSR medium should be 4 times the volume of Accutase used. Gently pipette the mixture up and down 4–6 times to break down the large clumps and transfer to a 15 mL conical tube.
7. Centrifuge the cell suspension at 200–300× *g* at room temperature for 5 min. During this time, add 4 μL ROCK inhibitor to 4 mL complete mTeSR medium to make the resuspension medium.
8. Aspirate the supernatant carefully, without removing the cell pellet, and gently resuspend the cells in the mTeSR/ROCK inhibitor resuspension medium.
9. hPSCs can be split in every passage using 1 in 4–6 splits (i.e., cell aggregates from 1 well will be plated into 4–6 wells). However, this can be varied in different iPSCs and is best determined by each investigator.
10. Prepare the appropriate volume of complete mTeSR medium in a separate 15 mL conical tube with ROCK inhibitor.
11. Aspirate the Matrigel coating solution from the desired plate or flask and add the appropriate volume of complete mTeSR medium/ROCK inhibitor without scratching the coated surface of the plate or flask.
12. Gently add the appropriate volume of resuspended cells to the wall of the plate or flask and incubate the cells in 37 °C at 5% CO_2_. When placing plates or flasks in the incubator, make sure to distribute the cells evenly by moving the plate or flask in an up-and-down and side-to-side motion.
13. Expand the cells as described above in Section 3.2.1.

### 3.3. Differentiation of hPSCs toward Skeletal Muscle Progenitors

#### 3.3.1. Stage I: Mesoderm Induction (Day 0–4)

**Figure 1:**
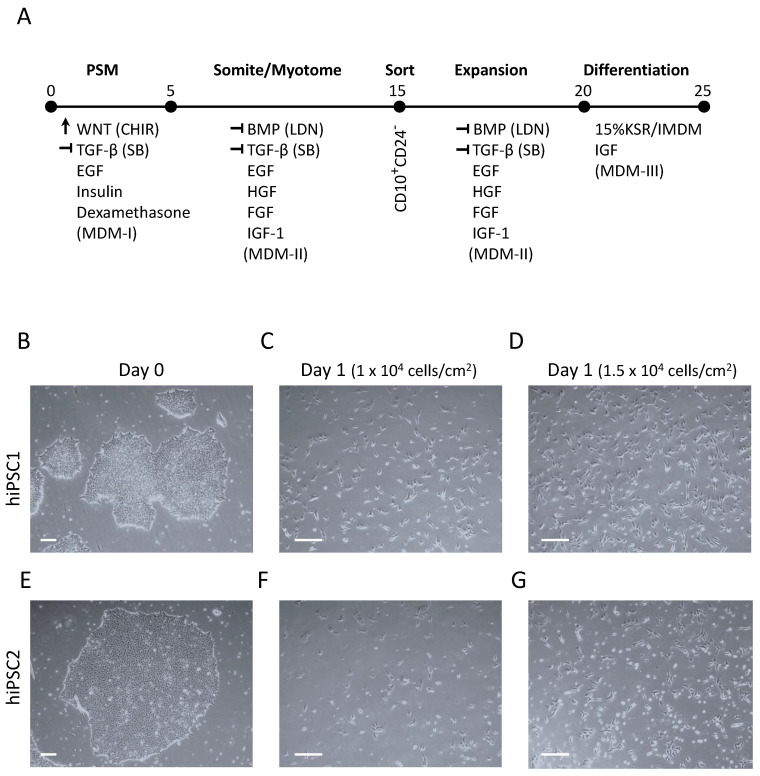
Protocol timeline and the effect of initial cell plating density on cell survival at Day 1 of differentiation. (**A**) Schematic figure demonstrating important stages of differentiation protocol. Important signaling molecules and growth factors for each stage of differentiation are listed. (**B–G**) Demonstration of the effect of initial cell density on cell survival after 1 day of differentiation. Upper row (iPSC1, **B–D**) demonstrates a control human iPSC line with acceptable cell survival at both tested densities. Lower row (iPSC2, **E–G**) indicates another iPSC line with reduced cell survival at 1 × 10^4^ cells/cm^2^ (**F**). Increasing initial plating density to 1.5 × 10^4^ cells/cm^2^ improved cell survival at Day 1 (**G**). Scale bars: 100 µm.

hPSCs should be passaged at least one time before starting myogenic differentiation protocol. In addition, colonies should be fully grown and ready for passage as demonstrated in Figure 1B,E (left images) (see Note 5).

**Figure 2:**
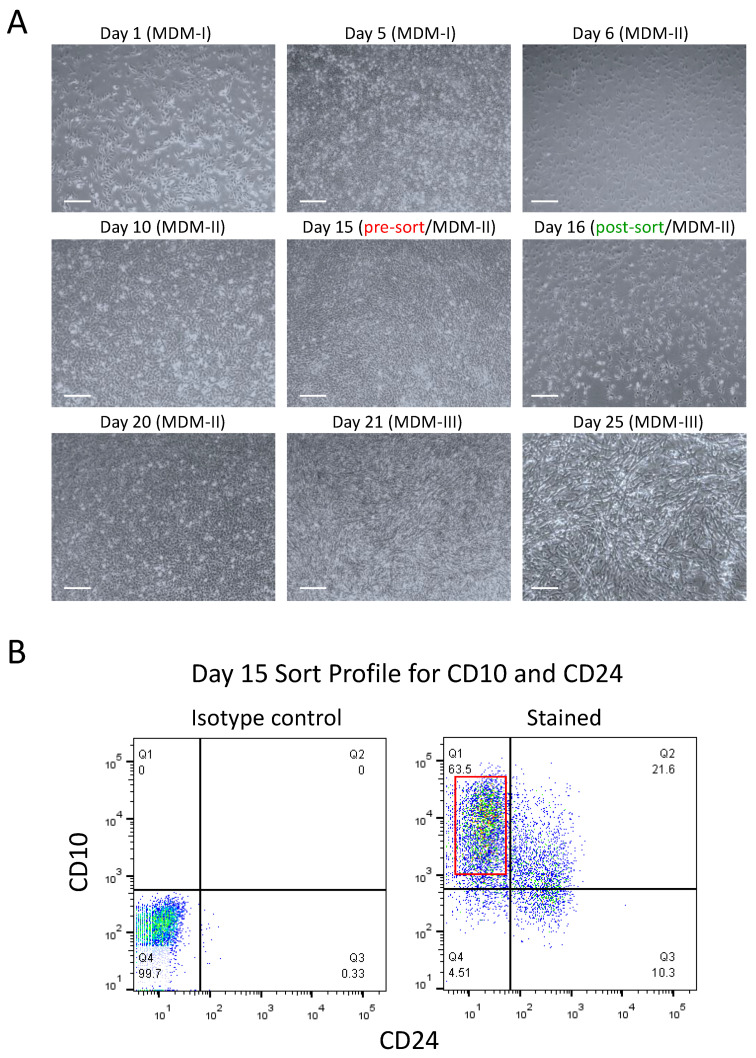
Morphology and sorting profile of hiPSC-derived myogenic progenitors during differentiation timeline. (**A**) Brightfield images demonstrate cell morphology density and transition at different time points during differentiation protocol. Cells become fully confluent at passage (Day 5), sort time point (Day 15) or before terminal differentiation (Day 20). Also note the transition of the cell morphology into spindle/myoblast shape at later stages after sort (Days 16–20) and their elongation into myotubes during terminal differentiation (Days 21–25). Scale bars: 100 µm. (**B**) Dot plots indicate proper differentiation of the cells at day 15 with formation of a distinct CD10^+^CD24^−^ cell population (marked with red box). Based on the iPSC type (healthy or patient derived), this percentage might be varied from 40–65% in an ideal differentiation condition.

1. Prior to differentiation, coat the desired plates or flasks with Matrigel, as described above (see Section 3.1). (see Note 6)
2. Prepare the Myogenic Differentiation Medium-I (MDM-I) (Table 3) using a 150 mL vacuum filtration flask (see Section 2.2 for more information about reagents). MDM-I should be wrapped in foil to protect the light-sensitive reagents used and can be stored in 4 °C for up to a week.
3. Harvest the cells with Accutase, as described above in steps 1–6 of Section 3.2.2.
4. After stopping the Accutase reaction and transferring the cells into a 15 mL conical tube, centrifuge at 200–300× *g* at room temperature for 5 min. During this time, prepare the resuspension medium by adding 4 μL ROCK inhibitor to 4 mL MDM-I.
5. Aspirate the supernatant, leaving the cell pellet, and gently resuspend cells in the 4 mL MDM-I/ROCK inhibitor resuspension medium by pipetting up and down 8–10 times to break cell aggregates into single cells for differentiation.
6. Count the cell number with a hemocytometer and calculate the volume of resuspended cells and MDM-I medium needed per well or flask at the density of 1 × 10^4^ cells/cm^2^ (Table 4). Prepare the appropriate volume of MDM-I medium in a separate 15 mL conical tube and add ROCK inhibitor.
7. Aspirate the Matrigel coating solution from the desired plate or flask and add the cell with appropriate volume of MDM-I medium/ROCK inhibitor without scratching the coated surface of the plate or flask.
8. Incubate the cells in 37 °C at 5% CO_2_. When placing plates or flasks in the incubator, make sure to distribute the cells evenly by moving the plate or flask in an up-and-down and side-to-side motion. Do not disturb the cells after even distribution until the following day (see Note 1).
9. Check that the cells are attached to the plate or flask and proliferating the next day (Day 1) using an inverted light microscope at 4× magnification. There may be a few cells that did not attach and are floating, but the majority of the cells should be attached (see Note 3). In an optimal condition, Day 1 differentiated cells should show homogeneous elongated morphology with 20–30% confluency, as demonstrated in Figure 1C,D. However, in the case of patient-derived iPSCs, Day 1 cell survival might be significantly low (Figure 1F). If so, increasing the initial plating density fixes the problem (Figure 1G). The appropriate cell density should be optimized based on each iPSC clone.
10. Change the cell culture medium by aspirating the used medium and adding fresh medium (MDM-I without ROCK inhibitor) to the wall of the plate or flask to prevent detachment of the proliferating cells. The cell culture medium (MDM-I without ROCK inhibitor) should be changed every other day after Day 1, (Days 1, 3) until the cells are ready to be passaged to Stage II, as demonstrated in Figure 2A (Day 5). In case the cells are proliferating rapidly and the medium is turning yellow, it may be necessary to change the medium every day.

#### 3.3.2. Stage II, Somite/myotome induction (Day 5–14)

At Day 5 of the differentiation time course, hiPSC-derived cells should be at 90–100% confluency (Figure 2A, Day 5) and ready to be passaged. (see Note 7). During this stage, cell morphology is changing to flat epithelial shape, and they distribute evenly without any colony formation, similar to monolayer somatic cells in culture.

1. Prior to passaging the cells and starting Stage II of the time course differentiation, coat the desired plates or flasks with Matrigel, as detailed above (see Section 3.1) (see Note 8).
2. Prepare the Myogenic Differentiation Medium-II (MDM-II) (Table 5) using a 150 mL vacuum filtration flask (see Section 2.2 for more information about reagents). MDM-II should be wrapped in foil to protect the light-sensitive ingredients used. MDM-II can be stored in 4 °C for up to a week.
3. Harvest the cells with Accutase, as described above in steps 1–6 of Section 3.2.2. Proceed to passage with similar cell density (1 × 10^4^ cells/cm^2^), as described in steps 4–8 of Section 3.3.1. Switch the medium to MDM-II. (see Note 9)
4. Check the cells the next day (Day 6) using an inverted light microscope at 4× magnification. There may be a few cells that did not attach and are floating, but the majority of the cells should be attached (Figure 2A, Day 6). (see Notes 2 and 3). Change the cell culture medium by aspirating the used medium and adding fresh medium (MDM-II without ROCK inhibitor) to the wall of the plate or flask. The cell culture medium (MDM-II without ROCK inhibitor) should be changed every other day until the cells are ready to be sorted (Day 15), but if the cells are proliferating rapidly and the medium is turning yellow, it may be necessary to change the medium every day. For reference, brightfield images of a typical culture have been demonstrated in Figure 2A throughout the differentiation time course.

#### 3.3.3. Cell Sorting and Expansion of Skeletal Myogenic Progenitors (Day 15–19)

At Day 15, cells are stained for CD10 and CD24 surface markers to the purify skeletal myogenic progenitors from the unwanted cells using flow cytometry (see Note 10).

1. Prior to sorting hPSCs, coat the desired plates or flasks with Matrigel to be used after cell sorting, as detailed above (see Section 3.1).
2. Warm FACS staining buffer (see Section 2.3) and Accutase at room temperature for about 30 min to 1 h.
3. Aspirate the used medium from the plate or flask and add the same volume of PBS to wash out any leftover MDM-II medium.
4. Aspirate the PBS wash and add enough Accutase to cover the surface of the plate or flask. Tilt the plate or flask to ensure complete coverage. Refer to Table 2 for the suggested volume of Accutase to add for each type of plate or flask.
5. Incubate the Accutase and cells in a 37 °C incubator for 3 min. After incubation, tap the plate or flask to make sure the cells have detached from the surface. If tapping does not seem to detach the cells, incubate for another minute. We do not recommend incubating for more than 5 min total because prolonged enzymatic reaction may be detrimental to the cells.
6. After removing the cells from the incubator, transfer the cells to a 15 mL conical tube and stop the enzymatic reaction by mixing the cells well with an appropriate amount of complete medium (4× the volume of Accutase used). Ensure cell aggregates are broken down into single cells by pipetting up and down 8–10 times.
7. Centrifuge the cell suspension at 200–300× *g* at room temperature for 5 min. This concludes the first wash of the cells. Prepare a bucket of ice for storage of cells in later steps.
8. Aspirate the supernatant and resuspend cells in 2 mL of FACS staining buffer. Mix well by pipetting up and down to ensure cells are broken down into single cells.
9. Transfer the resuspended cells to a FACS cell strainer capped tube to allow large cell clumps to be filtered out.
10. Remove most of the filtered cells (leaving 50–100 μL, in the FACS cell strainer capped tube) and transfer into a labelled 15 mL conical tube for staining with antibodies. The remaining cells left in the FACS cell strainer capped tube can be used for nonstained and isotype-stained controls to set up sort settings and adjust the gates.
11. For the second wash, thoroughly mix the filtered cells in the 15 mL conical tube with 10 mL of FACS staining buffer.
12. Centrifuge this cell suspension at 200–300× *g* at room temperature for 5 min. This concludes the second wash of the cells.
13. Aspirate the supernatant and resuspend cells in 100 μL of FACS staining buffer per 1 × 10^6^ cells. Add 1 μL of primary blocking antibody per 1 × 10^6^ cells (see Section 2.3) and mix well by pipetting up and down 4–6 times. Store on ice for 5 min.
14. Add 1 μL of each antibody per 1 × 10^6^ cells (CD10 and CD24, see Section 2.3), and mix well. Wrap the tube in foil to protect the light-sensitive antibodies and incubate on ice for 20 min (see Note 11).
15. Fluorochrome for CD10 and CD24 can be selected based on investigator’s preference (such as APC, PE, FITC, etc.), as long as the same clone of the primary antibody is used.
16. After incubation, add 10 mL of FACS staining buffer and mix well to wash off the antibodies.
17. Centrifuge the stained cells at 200–300× *g* at room temperature for 5 min.
18. Aspirate the supernatant and resuspend the stained cells in 0.5 mL of FACS sorting buffer with DAPI (see Section 2.3) per 1 × 10^6^ cells. Wrap the tube in foil to protect the stained cells from light and store on ice.
19. Sort CD10^+^CD24^−^ cells (to isolate skeletal myogenic progenitors) using a flow cytometry system (such as BD FACSAria II Flow Cytometer, BD Sciences).
20. Cells should be sorted into a 15 mL conical tube containing 4 mL of cold MDM-II medium. In a typical successful differentiation using control iPSCs from healthy individuals, the CD10^+^CD24^−^ fraction is a well-separated population and around 45–65% of the total cells, as demonstrated in Figure 2B. In iPSCs derived from patients with severe types pf muscular dystrophies, this fraction might be significantly less (30–45%).
21. After sorting, centrifuge the sorted cell population at 200–300× *g* at room temperature for 5 min. During this time, prepare the resuspension medium by adding ROCK inhibitor to MDM-II.
22. Aspirate the supernatant and resuspend cells in the 1 mL resuspension medium. Calculate the number of the cells. We recommend seeding the cells at a higher cell density (2 × 10^4^ cells/cm^2^) to improve cell proliferation (Table 6):
23. Prepare the additional resuspension medium by adding ROCK inhibitor to the calculated volume of MDM-II.
24. Aspirate the excess Matrigel coating solution from the flask or plate and add sorted cells (resuspended in appropriate volume of MDM-II with ROCK inhibitor, Table 6) to the wall of the plate or flask.
25. Ensure the cells have been evenly distributed across the plate or flask by moving it in a straight side-to-side and up-and-down motion when placing them into the incubator. Allow the cells to attach to the bottom of the plate or flask by incubating it at 37 °C at 5% CO_2_ overnight.
26. Check the cells the next day (Figure 2A, Day 16) using an inverted light microscope at 4× magnification. The majority of the cells should be attached and proliferating. (see Notes 2 and 3). Change the cell culture medium by aspirating the used medium and adding fresh medium (MDM-II without ROCK inhibitor) to the wall of the plate or flask. The cell culture medium (MDM-II without ROCK inhibitor) should be changed every other day until the cells are at 90–100% confluency. However, if the cells are proliferating rapidly and the medium is turning yellow, it may be necessary to change the medium every day.
27. Cells can be passaged after attaining maximal confluency (80–90% confluency after 3–5 days) and expanding as needed, up to a few passages (1–5) using the same plating density. Longer passages might reduce terminal myotube differentiation.

#### 3.3.4. Stage III: Terminal Differentiation into Myotubes (Day 20–25)

Expanded myogenic progenitors at passages 1–5 can be differentiated into myotubes after attaining 100% confluency in MDM-II, as demonstrated in Figure 2A (Day 20). (see Note 12)

**Figure 3:**
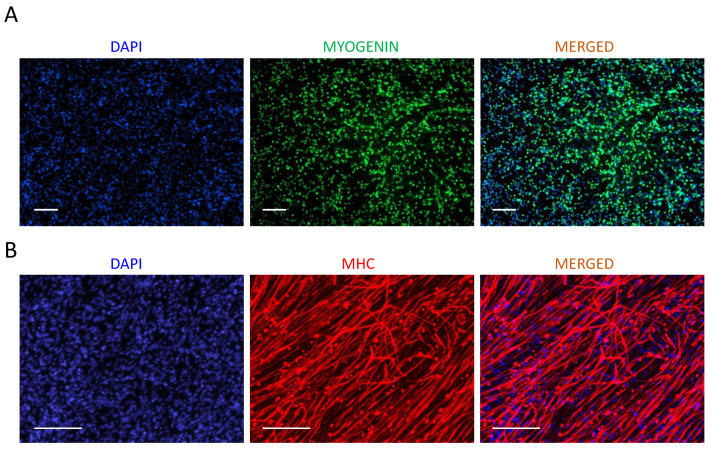
Myotube formation of hiPSC-derived sorted myogenic cells at terminal stage of differentiation. (**A**) Immunostaining for MYOGENIN (green) demonstrates uniform nuclear expression of the marker in terminally differentiated cells at the last time point. Scale bars: 100 µm. (**B**) Immunostaining for myosin heavy chain/MHC (red) demonstrates formation of elongated myotubes at the end of terminal stage. Scale bars: 100 µm.

1. Prepare the Myogenic Differentiation Medium-III (MDM-III) (Table 7) using a 50 mL conical tube and syringe filter system (see Section 2.4 for more information about reagents). MDM-III should be wrapped in foil to protect the light-sensitive reagents used and can be stored at 4 °C for up to a week.
2. Aspirate the used medium from the plate or flask and wash it twice with the same volume of PBS to wash out any leftover MDM-II medium.
3. Gently add MDM-III medium to the wall of the plate or flask to avoid detaching the cells. Refer to Table 6 for the total volume of medium needed for each type of plate or flask. Place the cells back into the incubator and monitor daily for cell elongation and myotube formation.
4. Feed the cells by replacing half of the used medium every day (see Note 13).
5. After 2–5 days of differentiation in MDM-III medium, myotubes are well formed in the culture and can be used for characterization by immunostaining (Figure 2A Days 21, 25). Differentiation time might vary among different iPSCs and should be monitored by daily microscopic evaluation of the plates for the appropriate formation and maturation of myotubes during this phase. Keeping the cells longer than this period might result in the detachment of the myotubes and their gradual loss. Therefore, the appropriate differentiation time should be optimized based on each hPSC clone.
6. To fix the cells, aspirate the used medium, wash once with PBS and incubate in fresh 4% PFA (see Section 2.5) for 15 min at room temperature.
7. Aspirate the PFA and wash with PBS twice. The fixed cell can be used for immunostaining for terminal differentiation markers [19,20] such as myogenin or myosin heavy chain (MHC) to characterize myotubes (Figure 3A,B). Alternatively, fixed plates can be kept in PBS at 4 °C for future staining up to a few weeks.

## 4. Notes

1. After plating at Day 0, iPSC clumps need some time to adhere to the plate. Therefore, they should not be disturbed during the first 12 h.
2. The day after seeding, most cells should be attached to the Matrigel-coated plate or flask. If there are a significant number of floating dead cells (>40%), the quality of the iPSCs may not be optimal. This might happen when working with patient-derived iPSCs. Therefore, we recommend restarting the experiment with a new batch of cells at lower passages. In addition, the initial plating density in these cases should be optimized to 2–3× to improve initial culture establishment.
3. ROCK inhibitor used in Day 0 changes the cell morphology to fibroblastic/elongated appearance. This is normal and after removal of ROCK inhibitor in Day 1, the cell morphology gradually shifts back to normal.
4. When tapping the plate or flask after the cells are incubated with Accutase, a thin film should be observed to detach from the surface of the plate or flask containing cell colonies. If tapping does not seem to detach the cells, incubate for another minute and tap again. We do not recommend incubating for more than 5 min total because prolonged enzymatic reaction may be detrimental to the cells.
5. After thawing, hPSCs need time to recover and reach their maximal proliferation rate, so waiting for at least one passage is necessary for formation of quality cells before starting the differentiation time course. If the cells do not seem to be proliferating as expected, we recommend thawing a lower passage of iPSCs.
6. For Stage I of the differentiation time course, we recommend using at least a 6-well plate to ensure that there are enough cells for later passaging into a T25 or T75 flask for Stage II.
7. If the cells are not 90–100% confluent after 5 days in MDM-I, we recommend harvesting and seeding the cells at a higher density (1.5–2 × 10^4^ cells/cm^2^) for stage II (in MDM-II).
8. We recommend using either a T25 or T75 flask for the beginning of Stage II, to ensure appropriate cell expansion needed for sorting.
9. Passaging the hPSCs and changing to MDM-II has to be performed at Day 5. Do not disturb the cells after passaging at Day 5 for at least 12 h to ensure appropriate cell adhesion to the Matrigel.
10. At Day 10 of the differentiation time course, differentiated hPSCs should be at 90–100% confluency. At this point, do not passage and continue to change MDM-II every other day to allow for further expansion until Day 15. At Day 15, the cell culture should look very dense and packed with cells. This condition is optimal and allows for maximal induction of skeletal myogenic progenitors ready for sort.
11. To protect the stock solutions of antibodies from degrading, ensure they are only removed from 4 °C storage during this step. They should be immediately returned to 4 °C after use.
12. For terminal differentiation of myogenic progenitors into multinucleated myotubes, a high cell density is imperative. Thus, the cells should reach 100% confluency prior to switching to MDM-III; otherwise, the cells may not fully differentiate into multinucleated myotubes and may detach from the plate or flask before the end of Stage III.
13. During terminal differentiation, as the cells are forming myotubes, cell adhesion to the surface of the plate or flask may be weakened. Therefore, be very gentle when changing the medium. Observe the peripheral regions of the flask to look for signs of detachment. Changing half of the medium every day may not be necessary if the culture and medium look good.

## Figures and Tables

**Table 1:** Volume of Matrigel solution needed per plate or flask.

Plate (per well) or Flask	Volume of Matrigel Mixture (mL)
24-well plate	0.3 mL per well
12-well plate	0.5 mL per well
6-well plate	1 mL per well
T25 flask	2.5 mL
T75 flask	7.5 mL

**Table 2:** Volume of Accutase needed per plate or flask.

Plate (per well) or Flask	Volume of Accutase (mL)
24-well plate	0.13 mL
12-well plate	0.25 mL
6-well plate	0.5 mL
T25 flask	1 mL
T75 flask	3 mL

**Table 3:** Reagents needed to make 100 mL of MDM-I.

Reagent	Volume (Total Volume 100 mL)	Final Concentration or %
IMDM base medium	93 mL	93%
Horse serum	5 mL	5%
3 mM CHIR99021	100 μL	3 μM
2 mM SB431542	100 μL	2 μM
10 μg/mL EGF	100 μL	10 ng/mL
10 mg/mL Insulin	100 μL	10 μg/mL
0.8 mg/mL Dexamethasone	50 μL	0.4 μg/mL
200 mM L-ascorbic acid	100 μL	200 μM
GlutaMAX	1 mL	1%
Pen Strep	1 mL	1%

**Table 4:** Number of hPSCs to be seeded per plate or flask.

Plate (per well) or Flask	Number of hPSCs	Total Volume (mL)
24-well plate	2.5 × 10^4^	0.5 mL
12-well plate	5 × 10^4^	1 mL
6-well plate	1 × 10^5^	2 mL
T25 flask	2.5 × 10^5^	5 mL
T75 flask	7.5 × 10^5^	15 mL

**Table 5:** Reagents needed to make 100 mL of MDM-II.

Reagent	Volume (Total Volume 100 mL)	Final Concentration or %
IMDM base medium	93 mL	93%
Horse serum	5 mL	5%
10 mg/mL Insulin	100 μL	10 μg/mL
10 μg/mL EGF	100 μL	10 ng/mL
20 μg/mL HGF	100 μL	20 ng/mL
20 μg/mL FGF	100 μL	20 ng/mL
10 μg/mL IGF-1	100 μL	10 ng/mL
2 mM SB 431542	100 μL	2 μM
0.5 mM LDN193189	100 μL	0.5 μM
200 mM L-ascorbic acid	100 μL	200 μM
GlutaMAX	1 mL	1%
Pen Strep	1 mL	1%

**Table 6:** Number of sorted hPSCs (CD10^+^CD24^-^ myogenic progenitors) to be seeded per plate or flask.

Plate (per well) or Flask	Number of Sorted hPSCs	Total Volume (mL)
24-well plate	5 × 10^4^	0.5 mL
12-well plate	1 × 10^5^	1 mL
6-well plate	2 × 10^5^	2 mL
T25 flask	5 × 10^5^	5 mL
T75 flask	1.5 × 10^6^	15 mL

**Table 7:** Reagents needed to make 50 mL of MDM-III.

Reagent	Volume (Total Volume 50 mL)	Final Concentration or %
IMDM base medium	42 mL	84%
Knockout serum replacement	7.5 mL	15%
10 μg/mL IGF-1	50 μL	10 ng/mL
Pen Strep	0.5 mL	1%

## Data Availability

Not applicable.
